# The Importance of Sports Performance Factors and Training Contents From the Perspective of Futsal Coaches

**DOI:** 10.2478/hukin-2013-0055

**Published:** 2013-10-08

**Authors:** João Serrano, Shakib Shahidian, Jaime Sampaio, Nuno Leite

**Affiliations:** 1 School of Sciences and Technology, University of Évora, Portugal.; 2 Departament of Sport Sciences, Exercise and Heath, University of Trás-os-Montes and Alto Douro, Portugal.

**Keywords:** coaching, experience, futsal, performance, training

## Abstract

The aim of this study was to identify the importance assigned by futsal coaches with different education levels to the sports performance factors (technical, tactical, physical and psychological) and to the training contents. The sample was divided into three groups (novice: n=35, intermediate: n=42; and elite coaches: n=15) depending on the degree of specific education, coaching experience and the level of the teams trained. To achieve this goal, the coaches answered a questionnaire previously validated by specialists in sport sciences. The results showed significant differences between the novice and elite group in small-sided games, inferiority games, opposition and execution timing of the training and drill items. The analyses also showed significant differences between the novice and intermediate group in inferiority games and opposition of the training and drill items. Although, no differences were identified between groups for the remaining performance factors and training and drill items considered, the identified trends provide a baseline related to the knowledge that contributes to the development of expertise of futsal coaches.

## Introduction

In the last decade the topic of coach-athlete relationship has been one of the main themes of research and debate (Jowett and Wylleman, 2006). It is widely known that coaches play a critical role in the lives of young athletes and have the potential to influence, positively or negatively, their sporting experiences (Bruner et al., 2011). This is supported by the premises that positive results in sports are associated with the quality of this relation (Rhind and Jowett, 2010), with the capacity of the coaches to effectively promote the sports development of the athletes and its implications on the quality of sports training (Abraham et al., 2006; Gilbert et al., 2006; Martindale et al., 2007).

Coaching involves a central tenet of improving team or athlete performance that requires a cognitive activity to make decisions upon a multitude of dynamic situational factors (Jones et al., 2003). Due to the adverse and unstable conditions of their activity, coaches are required to possess the ability to make dynamic decisions, requiring strategic intervention plans, supported by an intensive activity of reflection, decision and implementation. However, research has shown that current formal education programs do not adequately prepare coaches for their task (Abraham and Collins, 1998; Nelson et al., 2006). The consensus is that the curriculum of formal education programs continues to be centred on a classroom–based approach, heavily taught along didactic lines where prescriptive teaching methods dominate, where supervised practice field is absent (Mesquita et al., 2010) and other competences such as problem solving, decision making and innovation are neglected (Leite et al., 2011). According to Jones et al. (2003) the process of becoming an expert coach is influenced much more by their interactive, situational coaching experiences, observations of peers and knowledge sharing with other coaches that any professional preparation programs. A new approach to coach education based on coaching contexts should be invented and implemented, in order that both coaches and athletes are exposed to relevant and optimum learning experiences (Mesquita et al., 2010). Nevertheless, in Portugal, the aims, context, practice and contents of each coach level in terms of providing education within participation as opposed to performance level coaching are not completely defined (Mesquita et al., 2010).

At present, it is known that an adequate training of the athletes, through long-term plans is the fundamental condition for the development of sports elites (Leite et al., 2009). Studies of expertise development have provided several models explaining progression from novice to expert, e.g. the Development Model for Sport Participation (Côté, 1999; Côté et al., 2003) or the Long-Term Athlete Development (Balyi, 2002; Stafford, 2005). These plans include a set of successive steps (initiation, orientation, specialization and high level performance) which are associated with a particular knowledge that the coach should have in order to act with competence in the global and highly complex process of sports training and preparation (Côté et al., 2003; Mesquita et al., 2010).

Therefore, reality proves that the evolution of the athlete’s performance requires better and improved knowledge on the part of the respective coach (Côté and Gilbert, 2009). Similarly to what has been suggested for players, coaches also should pass through several stages of development to attain the expertise level (Leite et al., 2011). The coaches with the highest level of training and more years of experience in the sport have spent more in their training and have, of course, greater competence in adapting the contents of the training to the stages of development of children and young people. However, these coaches will also have higher expectations of sporting success, so they seek professional challenges of a higher competitive level, with greater personal visibility and with an appropriate remuneration, which they may find in senior teams of professional championships and not at the recreational levels. It appears, therefore, that the initial stages of the players’ development are areas confined to the intervention and responsibility of less experienced coaches (level I) (Leite et al., 2011), but these are the levels in which young athletes are most sensitive to learning and development of their physical, technical, tactical and psychological capacities. According to Balyi (2002), experienced coaches should be stimulated to get involved in the initial stages of athletic development. This early involvement may contribute to successfully lengthen the athletes’ career and be beneficial to their long-term qualitative development (Cushion et al., 2003). What is implicit within the concept of coaching expertise is the concept of “added value” that an expert coach can bring to the development of skills (cognitive, motor or emotional) of the youth athletes. Therefore, the input of a quality coach could provide a structured environment that optimizes learning (Abraham and Collins, 1998; Côté et al., 2003).

The process of educating coaches requires the acquisition, consolidation and development of skills, with the contribution of courses for coaches and especially the experience acquired from participation in sports events, both as an athlete and as a coach, a process heavily dependent on the competitive level of their participants. Taking into consideration the issues discussed above, it is worthy to note that these previous experiences may influence and interfere in training concepts and exercise contents. However, while most studies have focused on coaches behaviour during training sessions and during competitions (Smith and Cushion, 2006), there is still a lack of studies examining the importance given by coaches to training and drill items (Leite et al., 2011). While the available literature suggests substantial differences from novice to expert coaches across team sports, there is a limited comprehensible explanation on coaches’ perceptions about the importance of training factors and drills used in athletes’ development (Leite et al., 2011). Thus, the aim of this work was to identify the importance assigned by futsal coaches with different coach education levels to the sports performance factors (technical, tactical, physical and psychological) and to the various components of the training and drill items. It is expected that these results will allow to prioritize the knowledge that contributes to the developmental process leading to coaching expertise in futsal.

## Material and Methods

### Participants

Ninety-two futsal coaches participated voluntarily in this study. All participants obtained their certificates through the national certification programs. The coach experience variable was defined based on the number of years of experience (Mesquita et al., 2010). Abraham et al. (2006) considered 10 years as the minimum time required to distinguish experienced coaches from the novice. Nevertheless, Côté and Gilbert (2009) recognized that the coaching experience is a multidimensional variable not fully characterized by the number of years of working as a coach, and thus the highest level of the team coached throughout the carrier was also considered in this study. The sample was divided into three groups according to education certificates, experience and the level of the teams: (i) the novice group, composed of coaches holding level I futsal coaching certificates (*n*=35, age 34.8±7.1 years, experience 5.9±3.7 years); (ii) the intermediate group, composed of coaches with level II or III (*n* = 42, age 38.6±7.1 years, experience 8.1±3.0 years); (iii) the elite group, composed of coaches with level III or IV (*n* = 15, coaches of national champion teams or national teams, age 46.1±6.8 years; experience 19.9±7.5 years). This elite group was composed of eight Portuguese, five Spanish and two Brazilian coaches.

Forty per cent of the novice group was working with teams at the initial stages of the players’ development; 43% was working with senior teams competing at district championships and 7% was working with senior teams competing at the national championships. In the intermediate group, 36% of coaches was working with teams at the initial stages of the athletes’ development; 21% was working with teams competing at district championships; 31% was working with senior teams competing at the national championships; 12% was not coaching any team. In the elite group, coaches were mostly working with senior national teams (93%) and only 7% was working with teams at the initial stages of the players’ development. The study protocol followed the guidelines stated in the Declaration of Helsinki and was approved on November 20^th^ 2012 by the Ethics Commission of the Research Centre for Human Heath and Welfare of University of Évora, Portugal.

### Measures

The opinion of the coaches was measured using a questionnaire for basketball coaches previously used and validated by Leite et al. (2011). This questionnaire was adjusted to futsal and its validity was re-inspected by two futsal coaches with the highest level of training and with more than 20 years of coaching experience. The contacts of these coaches were provided by the Portuguese futsal team coach and the questionnaires were sent by email with a text explaining both the objectives of the study, and the details of the form. The questionnaire was translated into Spanish before being sent to the Spanish coaches. All the questions posed by the coaches in relation to filling the questionnaire were answered by email.

The questionnaire was composed of two parts: the first part covered the biographic information of the sample (age, gender and nationality). The second part of the questionnaire focused on the futsal training and drill items that they considered to be of greater relevance. To this effect, the coaches replied to 27 questions divided into five groups, namely (i) 4 technical-related factors: *individual technique* (dribbling, passing and shooting); *movements without ball* (ready stance, running, changing direction/speed) and *with ball* (parallels, diagonals, overlapping) and *defensive movements* (defensive position, swapping, covering); (ii) 8 tactical-related factors: *small-sided games* (1vs1, 2vs2, 3vs3, 4vs4), *offensive/defensive superiority/inferiority games* with goalkeeper (GK) (1vs0+GK, 2vs1+GK, 3vs2+GK, 4vs3+GK, 5vs4+GK), *match* (GK+4vs4+GK), *offense, defence, offense/defence transitions and defence/offense transitions*; (iii) 2 motor-related factors: *conditioning* (strength, endurance and flexibility) and *coordination* (agility, balance, coordination and speed); (iv) the psychological factors (teamwork, self-confidence, *decision-making*, leadership, resistance to stressful situations, etc.); (v) twelve training and drill items used in futsal: *execution technique, repetition, length, enjoyment, cooperation/opposition, competition, space, speed and timing of execution, decision-making* and *formal match*.

The answers were selected by the coaches from a set of options using a 5-point Likert scale (1 = rarely present in drills used in training sessions: 0–20% of the drills; 2 = unusually present in drills used in training sessions: 21 to 40%; 3 = present in drills used in training sessions: 41–60%; 4 = frequently present in drills used in training sessions: 61–80%; 5 = always present in drills used in training sessions: 81–100%).

### Analysis

The data from the first part of the questionnaire were processed in a spreadsheet to obtain the average and standard deviation of the answers in each group. The data of the second part of questionnaire were analyzed through oneway ANOVA and post-hoc multiple comparisons were done using the Tukey HSD test. The statistical analyses were performed using SPSS software release 16.0 (SPSS Inc., Chicago, IL) and significance was set at *p* ≤ 0.05.

## Results

Based on the results from the sports performance factors it was possible to identify similarities between groups concerning technical, physical and psychological capacities (Table 1). In tactical factors there were significant differences between the novice and elite group in two types of exercises: the small-sided games (p<0.01) and the inferiority games (p<0.05, Table 1), with the elite group showing greater preference for these tasks. Also, in case of tactical exercises, while the coaches with lower education levels (novice and intermediate groups) preferred match tasks, the coaches with higher education considered the match as less important, and gave preference to small-sided games. For elite coaches, the small-sided games, the superiority/inferiority games, the offense/defence transitions and defence/offense transitions are always present (81–100%), while the match, defence and offense exercises, were only used frequently (61–80%).

In training and drill items (Table 2), there were significant differences between the coaches groups in opposition, with differences between the novice and elite group (p<0.01) and between the novice and intermediate group (p<0.01), showing that the coaches with the higher education gave higher importance to this content. Additionally, there were differences in execution timing, with significant differences between the novice and elite group (p<0.05, Table 2).

## Discussion

The aim of this work was to understand how futsal coaches with different education levels evaluate the importance of training contents and sports performance factors. It was expected that these results would allow to prioritize the knowledge that contributes to the development of processes leading to coaching *expertise* in futsal. Available study results in basketball using similar methods identified that coaches of lower education levels (novice and intermediate) considered the technical factors and the coordination factors to be more important, while more experienced coaches (elite) considered tactical and physical conditioning factors more significant (Leite et al., 2011). These trends may indicate that the progression in coaches’ education goes from the technical and analytical-based training to the tactical and integrated-based training.

Unlike the results obtained by Leite et al. (2011) with basketball coaches, no differences were observed between the futsal coach groups in technical-related exercises (Table 1). The development of fundamental technical items, both defensive and offensive, at the first stages of development can be fundamental in coordinative training and in taking advantage of critical learning periods for basic sports techniques (Leite et al., 2011). The absence of differences between groups found in this study regarding technical items can be sustained by recent tendencies in approaching and teaching futsal. Sanz and Guerrero (2005) value more the adaptation to the context and unpredictability created for the adversaries than the gesture itself. Therefore, the technical implementation needs to be precise in order to be efficient, but it must also include mental (perception - analysis - decision) and motor execution. In fact, teaching futsal techniques should be done using global resources, favouring the improvement of the skills needed in the game development (Turner and Martinek, 1995).

Similarly, we found no differences between groups with regard to physical and psychological factors. These results do not confirm previous findings (Leite et al., 2011). These authors identified that more experienced coaches dedicated more time to *conditioning* (strength, speed or endurance). On the other hand, they also concluded that less experienced coaches, usually involved in the initial stages of athletic development (Mesquita et al., 2010), should take advantage of the optimal windows of trainability of *coordination* (Balyi, 2002).

Given the fact that futsal is a team sport, where collaboration and opposition occur in a continuous interaction, a great variability of unpredictable situations is created, with an alternation between offense and defence, which requires different physical and psychological behaviour from the athletes. Training should not only produce physiological response to each game situation, but must also develop a behaviour that can be transferred to competition, integrating all the qualities and factors that improve the capacity to play (Sanz and Guerrero, 2005). Several studies suggest that besides adjusting the physical loads to players’ maturational age, tasks proposed during practice should include technical, tactical and psychological factors (Memmert and Roth, 2007). At present, training is likely to include the psychological dimension variables of sports performance, such as motivation, concentration, control at the level of activation or confidence in one’s own capacities and resources (Sanz and Guerrero, 2005). Based on the assumption that the player is undergoing a process of continuous adaptation, the coach must provide him with learning conditions that maximize all of his resources.

Regarding tactical factors, significant differences were identified between novice and elite coaches in the small-sided games and inferiority games, with a clear preference of the elite group coaches for this type of exercises. The use of small-sided games and inferiority games should be regarded as a simplification of the real game (less players, adapted spaces). This is a way of improving technique and tactics, as well as increasing physiological and psychological capacities of players, since the intensity of the exercise can be manipulated, with implications at the level of decision-making and of the visual patterns (Vaeyens et al., 2007). Despite the crucial role of small-sided games in the coaching process, confirmed by the results of this study and well documented in recent scientific literature (Hill-Haas et al., 2008), very few studies are available on the importance of superiority or inferiority games. More importantly, literature is scarce when we try to establish a proper rationale between these items and the needs of futsal players’ development. Usually, defensive superiority games, such as 1vs2 or 2vs3, are complex game-like situations, which are related with the development of team defensive strategies and therefore, more specific to higher levels of competition (Leite et al., 2011). For these reasons, it is not difficult to understand the lower results obtained in this item, especially those corresponding to novice and intermediate coaches.

The results of this study seem to indicate that futsal, as a relatively recent sport, is at a development stage that is closer to the integrated training concept (Sanz and Guerrero, 2005), than to the traditional approaches to teaching/learning in team sports, such as basketball, primarily focused in the development of technique (Rink, 2001) and confirmed by the studies of Leite et al. (2011) with basketball coaches. Under the current methodology, futsal coaches seem to use more often drills that demand and highlight game intelligence (perception – analysis – decision) (Sanz and Guerrero, 2005). Leite et al. (2011) recognized that recent expansion of tactical-dominant models contributed to redefine team sports teaching/learning. In this particular approach, players are stimulated to develop tactical awareness and therefore, skill execution is permanently connected with the players’ performance in game-like situations. Consequently, the foundations of this model suggest that in early stages players should be confronted with tactical problems, helping them to develop their comprehension of the game and leading them to understand the need to optimize their skills in a game environment (Turner and Martinek, 1995).

The results of this study did not confirm the conclusions of Leite et al. (2011) about the different wide-ranging perceptions of basketball coaches, which led the authors to suggest the need for rethinking the models used by less skilled or inexperienced coaches when working with youth players. Futsal coaches, independently of their level of training and experience, attribute great importance to all sports performance factors. It can be assumed that the technical qualities, the tactical knowledge, the physical or psychological capacities are related and conditioned by each other.

With regard to training contents (Table 2), significant differences were observed between coach groups in two components: (i) opposition (between elite group and novice group, p<0.01; and between intermediate group and novice group, p<0.01); and (ii) execution timing (between elite group and novice group, p<0.05). These differences can be explained by the competitive level of the teams who the coaches were working with. While the coaches of the elite group are responsible for national selections or for international top teams, where the competitive pressure is high, approximately 40% of the coaches included in the novice group work with youth teams. While in the initial stages of the players’ development (where inexperienced coaches are more often involved) the opposition and the execution timing items are valuable but are not priorities, success in high-level competition depends greatly on the ability to beat the opponents, making better and quicker decisions throughout the game. Moreover, since the final score is almost the main indicator of success at this level, it is understandable that experienced coaches highly rank those drill items (Leite et al., 2011).

It is obvious that most coaches, independently of the level of training, consider decision-making to be more important and enjoyment to be less important. The greater importance attributed by the coaches in general to the decision-making component of the exercises as a fundamental component of game intelligence (perception – analysis – decision), associated to the significant greater use of small-sided games by the elite coaches group (Hill-Haas et al., 2008) demonstrates the evolution of futsal towards integrated training, as recommended by Sanz and Guerrero (2005). According to Abernethy et al. (2005) the importance of the decision-making component in the training exercises is justified by the need to expose the players to new situations of unpredictable context, and to develop their capacity for competition situations.

Thus, these results should benefit the debate among sports team coaches, in order to increase the quality of the sports training and promote an effective athletic development related with expert performers’ models (Leite et al., 2011). Selecting drills where game-like situations are more frequent, where cooperation and opposition occur in a dynamic interaction, stimulating the ability to execute skills at the right moment and encouraging tactical awareness, expressed by the constant need to make proper decisions, can benefit the development of the tools needed to achieve a higher level of performance (Leite et al., 2011). However, teaching players to make good and quick decisions is not an easy task (Turner and Martinek, 1995). What this study confirms is that, according to the importance attributed by experienced coaches, it is crucial for athletes to anticipate the stimulus (Leite et al., 2011).

For many years teaching and training in individual and team sports were based on the repetition of stereotyped movements. The partial progress (technical, tactical, physical) made by the players in analytical tasks seems to have little impact and transferability to the competition (Sanz and Guerrero, 2005). According to these authors, during the initial stages of teaching/learning, repetition can be important as a process of acquisition/consolidation of technical or tactical skills, and it is not surprising to observe in this study the occurrence of higher values of this component of the drills in the coaches with the lower levels of training than in coaches of the elite group.

The results of this study demonstrate the use of formal match as a component of training used always by the coaches, independently of their level of training and with average values that are very similar between the different groups of coaches (between 83 and 85%). In this context, all the attention is focused on game situations, providing an important space for decision making which subsequently or simultaneously, leads to the execution of the necessary technical and tactical elements within a game context (Turner and Martinek, 1995). The results of Wall and Côté (2007) indicate that for a 6 to 13 years age group, practice should focus primarily on game and on the different modified forms of the games, situations that promote learning and enjoyment. In the context of training, it is effectively essential to use the formal match as a resource for the development of an adequate conduct in competition (Sanz and Guerrero, 2005). According to these authors, in the context of high performance, the formal match, with or without adaptations to its structure or rules, must occupy a large part of the training sessions because it fulfils several functions of specific preparation for games of formal competition.

The results of this study reveal that, theoretically, less experienced coaches possess appropriate knowledge to lead and develop proper sports development of the young futsal players, a starting hypothesis of this work. It appears that the coach education process, which includes specific training courses, and specially the experience acquired from participation in sports events either as an athlete or as a coach, result in homogeneous training concepts, in the integration of the factors responsible for sports performance. Nevertheless, the lack of training courses with supervised field drills guided by experienced coaches (Mesquita et al., 2010) can jeopardize the practical application of this theoretical knowledge, with negative consequences at the level of the teaching/learning environment, and impact on the development of the athlete’s expertise. Thus, it would be interesting to extend this work through comparison of coaches with different levels of training and experience under real practical situations, seeking to verify the correspondence between the theoretical importance given to sports performance factors and their practical implementation in conducting the training. Other potential research lines are the evaluation of the influence of the methodologies used in football training on the conception of the futsal coaches or the influence of new technologies, f.ex. playing computer games, on understanding the game and on the concept of coaches.

## Figures and Tables

**Table 1:** Descriptive (mean ±standard deviation and confidence interval) and inferential statistics for technical, physical, psychological and tactical factors of sport performance

Factors	Items	Groups				
		Novice (n=35)	Intermediate (n=42)	Elite (n=15)	F (p)^*^	Tukey HSD (p)^*^
Technical 77.7 ±16.2 [74.4–81.1]	-Forward movements without ball: 68.3 ±25.2 [63.0–73.5]	66.3 ± 21.0 [59.1–73.5]	65.7 ± 27.0 [57.3–74.1]	80.0 ± 27.3 [64.9–95.1]	1.994 (0.142)	
	- Forward movements with ball: 81.5 ±17.1 [78.0–85.1]	78.9 ± 16.0 [73.3–84.4]	80.5 ± 17.4 [75.1–85.9]	90.7 ± 16.7 [81.4–99.9]	2.753 (0.069)	
	-Forward individual technique: 78.7 ±21.9 [74.1–83.2]	74.3 ± 22.5 [66.5–82.0]	80.0 ± 21.6 [73.3–86.7]	85.3 ± 20.7 [73.9–96.8]	1.482 (0.233)	
	-Basic defensive movements: 80.9 ±18.5 [77.0–84.7]	78.9 ± 19.4 [72.2–85.5]	81.0 ± 17.6 [75.5–86.5]	85.3 ± 19.2 [74.7–96.0]	0.639 (0.530)	

Physical 73.2 ±20.8 [68.8–77.5]	-Conditioning capacities: 77.2 ±23.3 [72.4–82.0]	71.4 ± 23.9 [63.2–79.6]	82.4 ± 19.9 [76.2–88.6]	76.0 ± 28.5 [60.2–91.8]	2.194 (0.117)	
	-Coordination capacities: 69.1 ±22.7 [64.4–73.8]	64.0 ± 23.2 [56.0–72.0]	70.5 ± 18.3 [64.8–76.2]	77.3 ± 30.1 [60.7–94.0]	1.997 (0.142)	

Psychological 78.7 ±22.3 [74.1–83.3]	-Psychological capacities: 78.7 ±22.3 [74.1–83.3]	73.7 ± 23.1 [65.8–81.7]	80.0 ± 23.0 [72.8–87.2]	86.7 ± 16.3 [77.6–95.7]	1.934 (0.151)	

Tactical 78.0 ±11.8 [75.6–80.5]	-Small sided games: 76.1 ±16.6 [72.7–79.5]	69.7 ± 17.1 [63.9–75.6]	78.1 ± 15.2 [73.4–82.8]	85.3 ± 14.1 [77.5–93.1]	5.781 (0.004)	b) p = 0.005;
	-Superiority games: 76.3 ±17.5 [72.7–79.9]	72.6 ± 17.5 [66.5–78.6]	76.7 ± 17.6 [71.2–82.2]	84.0 ± 15.5 [75.4–92.6]	2.315 (0.105)	
	-Inferiority games: 73.9 ±16.7 [70.5–77.4]	67.4 ± 13.8 [62.7–72.2]	76.7 ± 17.1 [71.4–82.0]	81.3 ± 17.7 [71.5–91.1]	5.111 (0.008)	b)p = 0.016;
	-Match (5x5): 79.3 ±19.5 [75.3–83.4]	80.6 ± 17.8 [74.5–86.7]	80.0 ± 20.7 [73.5–86.5]	74.7 ± 20.7 [63.2–86.1]	0.517 (0.598)	c) p= 0.035;
	-Offense: 79.1 ±16.7 [75.7–82.6]	76.0 ± 18.0 [69.8–82.2]	81.9 ± 15.2 [77.2–86.6]	78.7 ± 17.7 [68.9–88.5]	1.19 8 (0.307)	
	-Defence: 78.7 ±17.0 [75.2–82.2]	76.6 ± 18.5 [70.2–82.9]	80.5 ± 15.6 [75.6–85.3]	78.7 ± 17.7 [68.9–88.5]	0.499 (0.609)	
	-Offense – defence transitions: 79.3 ±17.1 [75.8–82.9]	76.6 ± 19.1 [70.0–83.1]	80.0 ± 16.5 [74.8–85.2]	84.0 ± 13.5 [76.5–91.5]	1.042 (0.357)	
	-Defence – offense transitions: 79.8 ±17.2 [76.2–83.3]	76.6 ± 19.1 [70.0–83.1]	81.0 ± 16.5 [75.8–86.1]	84.0 ± 13.5 [76.5–91.5]	1.168 (0.316)	

Values presented in the descriptive statistics are based on percentages; 0–20% - Rarely present; 21–40% - Unusually present; 41–60% - Present; 61–80% - Frequently present; 81–100% - Always present;

* Significant differences at p<0.05 at between: b) Expert and Novice; and c) Intermediate and Novice.

**Table 2:** Descriptive (mean ±standard deviation and confidence interval) and inferential statistics for training and drill items

Exercises	Items	Groups				
		Novice (n=35)	Intermediate (n=42)	Elite (n=15)	F (p)^*^	Tukey HSD (p)^*^
	-Cooperation: 81.1 ±18.6 [77.7–84.9]	76.0 ± 18.7 [69.6–82.4]	82.4 ± 18.8 [76.5–88.3]	89.3 ± 14.9 [81.1–97.6]	3.012 (0.054)	b) p=0.008;
	-Opposition: 79.1 ±17.0 [75.6–82.7]	72.6 ± 16.9 [66.8–78.4]	81.4 ± 16.2 [76.4–86.5]	88.0 ± 14.7 [79.8–96.2]	5.524 (0.005)	c) p=0.005;
Components 80.6 ±11.6 [78.2–83.0]	-Competition: 85.4 ±17.1 [81.9–89.0]	83.4 ± 18.5 [77.1–89.8]	85.2 ± 17.1 [79.9–90.6]	90.7 ± 12.8 [83.6–97.8]	0.949 (0.391)	
	-Repetition: 80.4 ±19.2 [76.5–84.4]	79.4 ± 18.5 [73.1–85.8]	82.4 ± 16.6 [77.2–87.6]	77.3 ± 27.1 [62.3–92.3]	0.454 (0.637)	
	-Execution speed: 81.7 ±16.7 [78.3–85.2]	78.3 ± 17.7 [72.2–84.4]	82.4 ± 16.1 [77.4–87.4]	88.0 ± 14.7 [79.8–96.2]	1.873 (0.160)	
	-Execution technique: 79.3 ±18.4 [75.5–83.2]	76.6 ± 19.7 [69.8–83.3]	80.5 ± 16.2 [75.4–85.5]	82.7 ± 21.2 [70.9–94.4]	0.718 (0.490)	
	-Length: 77.6 ±18.7 [73.7–81.5]	74.3 ± 19.1 [67.7–80.9]	78.6 ± 18.4 [72.8–84.3]	82.7 ± 18.3 [72.5–92.8]	1.159 (0.318)	b) p =0.036;
	-Execution timing: 81.1 ±17.6 [77.4–84.7]	76.0 ± 18.7 [69.6–82.4]	82.4 ± 17.2 [77.0–87.7]	89.3 ± 12.8 [82.2–96.4]	3.378 (0.039)	
	-Decision-making: 88.7 ±16.3 [85.3–92.1]	84.6 ± 18.8 [78.1–91.0]	90.5 ± 14.8 [85.9–95.1]	93.3 ± 12.3 [86.5–100.2]	2.017 (0.139)	
	-Space: 83.9 ±15.8 [80.7–87.2]	80.6 ± 16.4 [74.9–86.2]	84.8 ± 15.8 [79.8–89.7]	89.3 ± 12.8 [82.2–96.4]	1.765 (0.177)	
	-Game: 84.3 ±16.7 [80.9–87.8]	82.9 ± 19.5 [76.2–89.6]	85.2 ± 16.0 [80.3–90.2]	85.3 ± 11.9 [78.8–91.9]	0.221 (0.802)	
	-Enjoyment: 60.7 ±20.6 [56.2–64.9]	61.1 ± 20.0 [54.3–68.0]	59.5 ± 19.5 [53.4–65.6]	62.7 ± 26.0 [48.2–77.1]	0.141 (0.868)	

Values presented in the descriptive statistics are based on percentages; 0–20% - Rarely present; 21–40% - Unusually present; 41–60% - Present; 61–80% - Frequently present; 81–100% - Always present;

* Significant differences at p<0.05 at between: b) Expert and Novice; and c) Intermediate and Novice;
